# Socioemotional Goals and Definitions of Emotion Terms: A Qualitative Analysis Across Age

**DOI:** 10.1093/geroni/igab046.2846

**Published:** 2021-12-17

**Authors:** Irina Orlovsky, Rebecca Ready, Bruna Martins-Klein

**Affiliations:** 1 University of Massachusetts Amherst, Northampton, Massachusetts, United States; 2 University of Massachusetts Amherst, Amherst, Massachusetts, United States

## Abstract

Major theories of adult development posit that knowledge about emotion might evolve across the lifespan. Socioemotional Selectivity Theory (SST) and the Strength and Vulnerability Integration (SAVI) models imply that the manner in which older (OA) and younger adults (YA) conceptualize emotions may differ in valence, arousal, reference to social partners, time perspective, and the self. Quantitative accounts of age differences in conceptualizations of emotion-terms offer mixed support for theoretical expectations, but many predictions have yet to be tested qualitatively. In this study, 90 OA and 210 YA provided narrative descriptions of 11 (5 positive, 6 negative) emotion-terms. Responses were coded on valence, reference to self/others, and arousal. O/YA used similar synonyms to define emotion-terms. As predicted, YA used high arousal language in their definitions of negative (OR = 10.29, p = 0.018) and positive terms more than OA (i.e. Happy: OR = 1.27, p<0.001); OA referenced other persons such as family and friends (pos: OR = 0.13, p<0.001; neg: OA = 0.32, p=0.002) more than YA. Contrary to predictions, OA self-referenced more often than YA in positive (OR = 0.12, p=0.001) and negative definitions (OR= 0.11, p=0.004); this may be attributed to OA providing more situational examples in their responses than YA. Somewhat consistent with SAVI and SST, OA may reference high-arousal states less when conceptualizing emotions and associate their definitions more with social partners than YA. Future research should address OA greater use of situational examples when defining emotion terms, motivational factors and emotional impact of these age differences.

